# Signaling Networks of Activated Oncogenic and Altered Tumor Suppressor Genes in Head and Neck Cancer

**DOI:** 10.4172/2157-2518.S7-004

**Published:** 2013-08-05

**Authors:** Bin Yan, Robert Vander Broek, Anthony D Saleh, Arpita Mehta, Carter Van Waes, Zhong Chen

**Affiliations:** 1 Department of Biology, Hong Kong Baptist University, Kowloon, Hong Kong, China; 2 Tumor Biology Section, Head and Neck Surgery Branch, National Institute on Deafness and Other Communication Disorders, NIH, Bethesda, MD USA; 3 NIH Medical Research Scholars Program, Bethesda, MD USA

**Keywords:** Oncogenes, Tumor suppressor genes, Gene profiling, miRNAs, Genetic alterations, DNA methylation

## Abstract

Head and neck squamous cell carcinoma (HNSCC) arises from the upper aerodigestive tract and is the six most common cancers worldwide. HNSCC is associated with high morbidity and mortality, as standard surgery, radiation, and chemotherapy can cause significant disfigurement and only provide 5-year survival rates of ~50–60%. The heterogeneity of HNSCC subsets with different potentials for recurrence and metastasis challenges the traditional pathological classification system, thereby increasing demand for the development of new diagnostic, prognostic, and therapeutic tools based on global molecular signatures of HNSCC. Historically, using classical biological techniques, it has been extremely difficult and time-consuming to survey hundreds or thousands of genes in a given disease. However, the development of high throughput technologies and high-powered computation throughout the last two decades has enabled us to investigate hundreds or thousands of genes simultaneously. Using high throughput technologies, our laboratory has identified the gene signatures and protein networks, which significantly affect HNSCC malignant phenotypes, including TP53/p63/p73 family members, IL-1/TNF-β/NF-κB, PI3K/AKT/mTOR, IL-6/IL-6R/JAK/STAT3, EGFR/MAPK/AP1, HGF/cMET/EGR1, and TGFβ/TGFβR/TAK1/SMAD pathways. This review summarizes the results from high-throughput technological assays conducted on HNSCC samples, including microarray, DNA methylation, miRNA profiling, and protein array, using primarily experimental data and conclusions generated in our own laboratory. The use of bioinformatics and integrated analyses of data sets from different platforms, as well as meta-analysis of large datasets pulled from multiple publicly available studies, provided significantly higher statistical power to extract biologically relevant information. The data suggested that the heterogeneity of HNSCC genotype and phenotype are much more complex than we previously thought. Understanding of global molecular signatures and disease classification for specific subsets of HNSCC will be essential to provide accurate diagnoses for targeted therapy and personalized treatment, which is an important effort toward improving patient outcomes.

## Introduction

Head and Neck Squamous Cell Carcinoma (HNSCC) is the most common cancer that arises from epithelia of the upper aerodigestive tract, which includes the oral cavity, pharynx, and larynx [1]. There is an annual incidence of over 500,000 new cases of HNSCC worldwide, including 40,000 in the U.S. [1]. HNSCC of the oropharynx is associated with human papilloma virus (HPV) infection in ~25% of cases, while the remaining 75% are predominantly associated with tobacco use [1]. Standard therapies, which include surgery, radiation, and chemotherapy, provide 5-year survival rates of ~50–60% in U.S., making HNSCC one of the most difficult cancers to treat and a common cause of cancer related deaths [1]. Because of the heterogeneity of HNSCC subsets with different potentials for recurrence and metastasis, the traditional pathological classification of HNSCC exhibits limitations in clinical diagnosis and prognosis. As such, there is a tremendous demand for the development of new diagnostic, prognostic, and therapeutic tools based on the molecular signatures of HNSCC.

Cancer is a complex genetic disease driven by the accumulation of genetic alterations and functional defects in multiple pathways. Gene signatures that define malignant phenotypes are usually under the regulation of a few transcription factors, such as nuclear factor-kappaB (NF-κB) and tumor protein p53 (TP53). Previously, our laboratory discovered that the transcription factor NF-κB is aberrantly activated in HNSCC and serves to dysregulate a diverse repertoire of genes that promote cell proliferation, survival, inflammation, angiogenesis, tumorigenesis, and therapeutic resistance [2,3]. Our early studies have shown that constitutive activation of the NF-κB pathway through IL-1/TNF-α/IKK signaling and their downstream molecules contributes to tumorigenesis and metastasis in a murine Squamous Cell Carcinoma (SCC) model [4,5], which is consistent with observations in human HNSCC cell lines, patient serum and tumor specimens [6–13]. NF-κB regulated gene signatures are associated with the recurrent and metastatic human HNSCC genetic subtypes [14]. Tobacco and HPV, which are known etiologic factors of importance in HNSCC, have subsequently been implicated in the activation of NF-κB [15,16]. Using high throughput technologies of gene profiling, protein array and tissue array, we have identified the gene signatures and protein networks responsible for reciprocal crosstalk between NF-κB and TP53, two key factors which significantly affect HNSCC malignant phenotypes [17–20]. Further, we found that alternatively transcribed TP53 family members, such as ΔNp63 or TAp73 protein, orchestrate malignant phenotypes by forming protein or DNA binding complexes with NF-κB family members cREL and/or RELA. These complexes function as either repressors or synergistic agonists of TP53 and NF- κB activity [21–23]. Concurrently, using both classical biological and high throughput approaches, we and collaborators have characterized multiple additional signaling pathways that cross-talk with NF-κB and TP53 family members, including PI3K/AKT/mTOR [24,25], IL-6/IL-6R/JAK/STAT3 [26–28], EGFR/MAPK/AP1 [24,29,30], HGF/cMET/EGR1 [31–33], and TGFβ/TGFβR/TAK1/SMAD [34–37] pathways. Together, these pathways all contribute to the survival, migratory and angiogenic mechanisms which are important in the malignant phenotype of HNSCC.

In the past, the major challenge of identifying molecular signatures of HNSCC has been in overcoming the difficulties associated with surveying hundreds or thousands of genes or molecules, which is extremely time-consuming using classical biological techniques. However, in the last two decades, the advancement of high throughput technology and high-powered computation has enabled us to investigate hundreds or thousands of genes simultaneously. Consequently, we are now in a technological era with the potential for the explosive dissemination of knowledge and information.

## Microarray and Gene Expression Profiling of Tumor Lines

Alteration in expression profiles of oncogenes and tumor suppresser genes is fundamental to the classification of tumor phenotypes by stage, aggressiveness, heterogenic subsets, potential for recurrence and metastasis, and drug resistance [38]. Investigations of distinct gene profiling signatures reveal characteristics of tumor phenotypes in different cancer sub-populations, which leads to the identification of potential molecular biomarkers that are clinically useful for predicting prognosis and therapeutic efficacy [39,40]. Among the first groups to investigate molecular signatures of HNSCC, we pioneered the first generation of microarrays in relevant animal models and human cell lines.

Previously, we established a syngeneic and multiple-step murine SCC model in which the parental Pam 212 line exhibited low metastatic potential, while two cell lines derived from lymph node metastases, exhibited aggressive metastasis when reinoculated *in vivo* [41]. In this animal model, we found that the metastatic cell lines LY-2 and LY-8 displayed an increase in constitutive NF-κB activation and TNF-α inducible expression of proinflammatory cytokines, when compared with parental Pam 212 cells. The aberrant activation of NF-κB contributes to increased expression of proinflammatory cytokines during metastatic tumor progression [5]. These findings led us to identify changes in a broad gene expression profile of transformation and metastasis. mRNA differential display and cDNA array profiles enriched for cancer-associated genes were utilized to detect global expression differences among primary keratinocytes, parental Pam 212 cells and metastatic LY-2 cells [42]. We identified distinct malignant and metastatic gene signatures involved in growth and cell cycle (p21, p27, and cyclin D1), resistance and apoptosis (glutathione-S-transferase, cIAP-1/BIRC2, PEA-15, and Fas ligand), inflammation and angiogenesis [chemokine growth-regulated oncogene 1 (also called KC, human IL-8 homolog)], and signal transduction [c-Met, yes-associated protein (YAP), and syk]. Strikingly, 10 of 22 genes in the cluster expressed in metastases have been associated with activation of the NF-κB signal pathway. Subsequently, we showed that NF-κB-inducible cytokine Gro-1 was able to promote tumor growth, metastasis, and angiogenesis *in vivo* [43]. Many of these candidates have been validated as important oncogenic and tumor suppressor genes which contribute to HNSCC malignant and metastatic phenotype.

Next, we performed a cDNA microarray in a panel of HNSCC lines and showed that gene expression signatures of tumor subsets were related to NF-κB activation and/or deficient or mutated TP53. Bioinformatic analysis of the promoters and ontogeny of these clustered genes revealed two groups of HNSCC exhibiting distinct gene signatures: one set enriched for a higher prevalence of TP53 promoter binding motifs, and a second set enriched for injury response genes with NF-κB regulatory motifs. The results were confirmed with immunohistochemical staining, ChIP assays assessing promoter binding of NF-κB, and functional assays with siRNA mediated gene knockdown [18,20]. We concluded that NF-κB promotes cell survival and expression of a novel gene signature in HNSCC with deficient wildtype TP53, a subset previously associated with greater resistance to chemo-radio-therapy and worse prognosis. Thus, our early work using microarray profiling in murine tumor models and HNSCC cell lines revealed novel gene expression signatures that distinguished cancer cell subsets associated with NF-κB and/or TP53 activation status.

## Meta-Analysis of Gene Profiling in HNSCC Tissue Specimens

Genome-wide microarray technology has been in place for more than a decade. Over this period of time, massive microarray datasets have become available from a broad sample of HNSCC patient specimens representative of varying pathological conditions, including pre-malignant lesions, and primary, metastatic, and recurrent tumors [44,45]. A systemic study collected 63 published microarray datasets of HNSCC tissues and performed meta-analysis of gene sets [45]. These microarray studies cover hundreds of HNSCC tumor samples derived from a variety of anatomic sites such as oral cavity, pharynx, and larynx. Forty one of the 63 microarray datasets in this study analyzed primary tumor tissues vs. normal mucosa. Meta-analysis of these datasets has generated lists of gene sets that exhibit statistically significant differences between tumor and normal. For example, a list of 25 genes differentially expressed in primary tumors was identified in at least 9 of the 41 studies. These 25 genes are primarily involved in the biological processes of collagen metabolism, cell adhesion and migration, extracellular matrix (ECM)-receptor interactions, and inflammation. The strongest gene signature revealed 16 genes, which were consistently observed in more than 10 microarray datasets of HNSCC. Ten genes were overexpressed in the primary tumors compared to normal mucosal tissues, including *COL1A2, FN1, IFI6, ITGA6, MMP1, PLAU, POSTN, SPP1, SPARC* and *TNC*. Conversely, decreased expression of 6 genes, *ECM1, EMP1, KRT4, KRT13*, *MAL,* and *TGM3* was consistently observed in tumor specimens. In another analysis of 23 HNSCC gene expression profile studies, a panel of nine genes including *FN1, MMP1, PLAU, SPARC*, *IL1RN, KRT4, KRT13, MAL* and *TGM3, was identified as a set of* molecular markers with potential clinical diagnostic value [46]. These nine genes overlapped with the 16-gene panel from the previous meta-analysis, *and were validated in tumors and matched normal mucosa using qRT-PCR*. Each genes’ individual clinical relevance was subsequently evaluated according to statistical significance [46]. Notable among the genes in that panel, *MMP1* was consistently over-expressed in 13 of 41 microarray studies of tumor tissues from the meta-analysis [45], as well as in 9 of 23 microarrays in the second study, which was validated in 51 saliva samples from head and neck cancer patients [46]. Although these genes were identified in primary tumors, their functions are highly related to processes involved in metastasis, such as cell adhesion, motility and migration [47–49]. Consequently, these gene signatures could be tested in future clinical studies as candidate biomarkers in the primary tumor for predicting metastatic potential.

Similarly, a meta-analysis of 507 HNSCC tissue samples with different metastatic statuses was conducted on 20 published microarray studies [45]. A panel of 25 genes was identified to be differentially expressed in 3 or more studies of metastatic tumor tissues compared with non-metastatic tumor tissues. Importantly, in at least 4 studies, 5 genes upregulated in metastatic tissues are overlapped with genes observed to be upregulated in primary tumors. The overlapped panel of up-regulated genes includes *FN1, MMP1, PLAU, POSTN,* and *TNC.* Additionally, down-regulated *TGM3* was consistently found in 4 metastatic microarray studies [45]. These data further suggest that this gene signature in HNSCC primary tumors could serve as a predictor for metastatic disease. Several other studies utilized gene expression profiling to predict the clinical outcomes of HNSCC, such as the risk of recurrence and patient survival [50–54]. Overexpressed *MMP1* and *POSTN* were identified in both lymph node positive and recurrent tumors, revealing their roles in predicting HNSCC recurrence [54]. Extracellular matrix proteins *PLAU, SERPINE1* and *SPARC* were validated as prognostic markers for predicting overall survival of HNSCC patients [55]. In addition, Chung *et al.* defined a 75-gene list for determining disease recurrence in 60 HNSCC patients, and suggested the list as a prognostic biomarker of recurrence and molecular predictor of epithelial-to-mesenchymal (EMT) transition [53]. Consistent with our findings in murine tumor metastasis models and human HNSCC cell line models, Chung found a strong gene signature associated with NF-κB activation that may serve as a target for novel therapies for patients at high risk of standard-of-care treatment failure.

However, there are certain limitations to our interpretations of microarray studies which are caused by differing sample sizes and divergent sources, differing technologies or platforms employed, and differing data analysis methods with varying resolutions. This can lead to low consistency among multiple reports. These limitations can be addressed by integrating large numbers of datasets from multiple independent but related microarray studies. Meta-analysis of multiple large sample-sized microarrays of HNSCC tissues can enhance reliability and generalizability of the genome-wide expression profiles, which generates more precise and clinically useful biomarkers for HNSCC.

## DNA Methylation in HNSCC

One important regulatory mechanism of profoundly altered gene expression in cancer is through DNA methylation. As a type of epigenetic modification in the human genome, methylation is able to govern gene expression by modifying DNA without altering the sequence. It is commonly known that DNA methylation and its interplay with other epigenetic events in human cancer, such as histone modification, is crucial to the regulation of genome function through changing chromatin architecture [56]. For example, inactivation of certain tumor-suppressor genes occurs as a consequence of hypermethylation within the promoter regions. Recent advances using high throughput technologies make it possible to efficiently analyze epigenetic effects of DNA methylation on gene expression in human cancer [57–59], demonstrating a broad range of methylated genes in different cancer types [57,58,60,61]. The altered DNA methylation patterns in cancer have been proposed as candidates for the development of epigenetic biomarkers for early detection, diagnosis, prognosis, and therapeutic efficacy [57,61,62]. Experimentally, several methodologies have been developed to profile DNA methylation genome-wide, such as the use of methylation-specific endonucleases, bisulfite modification of unmethylated cytosines, and immunoprecipitation of methylated DNA fragments [63,64].

Silencing of tumor suppressor genes caused by the hypermethylation in HNSCC was proposed as an epigenetic mechanism that plays an important role in tumor initiation and progression [63–66]. Several represented genes, *TIMP3, p16* (*CDKN2A*)*, p14, MGMT, CDH1, RASSF1A* and *DAPK*, are highly sensitive to methylation-induced repression in HNSCC tumor and saliva samples, and are directly associated with tumor development [67–70]. In addition, a set of 15 candidate genes was tested for methylation status in HNSCC patients, of which *CDH1, p16, DAPK, hMLH1, MGMT, MST1*, *RARβ, RASSF2, RASSF5* were more significantly hypermethylated on the promoters in tumors than matched normal mucosal tissues [71]. Furthermore, in contrast to hypermethylation, global hypomethylation is also an aberrant epigenetic modification in cancer, which induces genomic instability and contributes to cell transformation [56,63,65].

Multiple groups performed genome-wide methylation profiling experiments covering four hundred HNSCC patient samples, and found that DNA methylation patterns are characterized by promoter hypermethylation and global hypomethylation [72–81]. We have summarized these 10 DNA methylation profile studies and have extracted the more frequently methylated genes in HNSCC patient samples [72–81]. Among those affected genes, *HOXA9* (*homeobox A9*) is the most frequently methylated, repeatedly showing promoter hypermethylation in 5 of the 10 HNSCC methylation profiling studies. The second most frequent site of promoter hypermethylation is on *EYA4*, which has been observed in 4 of the 10 methylation studies. In addition, we found 11 genes, *EPHA5, HS3ST2*, *SOX17*, *ADCYAP1, AIM2, CALCA, DCC, EMR3, HOXA11, HTR1B* and *MME*, were methylated in at least three HNSCC studies. Silencing of these genes was consistently observed in primary HNSCC when compared with normal mucosa, indicating the significance of epigenetic alterations in HNSCC pathogenesis. Together, these genes provide important clues for decoding HNSCC epigenomics and serve as potential targets for the discovery of diagnostic epigenetic biomarkers in HNSCC.

## Identification of Genetic Alterations through Large-Scale Exome Sequencing

Large-scale, massively parallel sequencing has provided great insight into signaling pathways, which accelerates our understanding of the biological mechanisms underlying HNSCC and how to better tailor treatment for patients. High-powered, high-throughput analyses of tumor DNA copy number variations and mutations through massively parallel sequencing have provided powerful information that can be used to better identify genetic drivers of HNSCC development and progression. In one recent study of the mutational landscape in HNSCC, matched tumor and whole blood samples from 92 HNSCC patients were subjected to whole-exome sequencing and hybrid capture analysis [82]. In addition to validating previously reported genetic alterations in HNSCC, such as *CCND1*, *MYC*, *EGFR*, *ERBB2*, and *CCNE1* amplifications, *CDKN2A* deletions, and *TP53*, *CDKN2A*, *HRAS*, *PTEN*, and *PIK3CA* mutations [38], this study implicated many genes which were previously unrecognized to be important players in HNSCC. Most notably, genes which regulate epidermal development and squamous differentiation, (e.g. *NOTCH1*, *NOTCH2*, *NOTCH3*, *IRF6*, and *TP63*), were observed to be mutated in over 30% of the samples. Other genes which mediate calcium-sensing (*RIMS2* and *PCLO*) and nuclear polarity (*SYNE1* and *SYNE2*), processes critical to squamous epithelial differentiation, also harbored mutations in subsets of 10–20% of the HNSCC samples studied. Besides the findings relevant to squamous differentiation, genes involved in apoptosis (*CASP8* and *DDX3X*) and regulation of gene expression (*PRDM9* and *EZH2*) were disrupted in 5–10% of cases.

In a second large-scale sequencing study conducted around the same time period, copy number analysis on 42 matched HNSCC tumor/normal pairs and sequencing of tumors from 32 patients was performed [83]. The findings complement the results of the study of 92 patient samples, in that *NOTCH1* was one of the most frequently mutated genes. Interestingly, nearly 40% of the mutations in *NOTCH1* were inactivating, which suggests that it may be acting as a tumor suppressor rather than an oncogene in HNSCC. The prevalence of mutations observed in an F-box protein family member, *FBXW7*, which targets *NOTCH1* for degradation, were also speculated to play a role in modulating the NOTCH pathway in HNSCC.

Both of the previously mentioned studies substantiated many of the genes which were already understood to be critical and representative of HNSCC. Taken together, these studies also indicate the importance of the dysregulation of terminal epithelial differentiation in HNSCC, a finding which had not been fully appreciated previously. Finally, in non-HPV-associated HNSCC, it became apparent that more than four times as many mutations occur in tumor suppressor pathways compared to oncogenes [83]. This is an important consideration in the development of novel targeted therapies for HNSCC, as most are currently directed at oncogenes.

## miRNA Profiling and Experimental Validation in HNSCC

MicroRNAs (miRNAs) comprise a highly conserved class of small RNA molecules (18–24 bp) that primarily bind to the 3′ UTR of mRNA molecules and either block translation or promote mRNA degradation. Alteration of miRNA gene expression due to genetic defects, such as DNA copy number variations or deregulation of miRNA expression, has been shown to contribute to carcinogenesis, including HNSCC. Several studies have reported global miRNA expression changes in HNSCC, using various samples sizes, anatomical sites, and profiling methodologies [84–87]. The most consistently overexpressed miRNA in HNSCC is miR-21, which has been reported to be transcriptionally activated by NF-κB and STAT3 [88–90]. Increased miR-21 has been reported to alter HNSCC cell survival, invasion, metastasis and resistance to chemotherapeutics [91]. The most frequently repressed miRNAs in HNSCC are the miRNA family members miR-99a, miR-100 [84], and miR-375 [92–95]. All three of these miRNAs have been shown to target IGF-1R in HNSCC [84,96], which is frequently overexpressed in many cancers [97]. Three other repressed microRNAs in HNSCC have also been reported to target mRNAs that we have identified as increased in our meta-analysis. miR-29c has been linked to p53 expression [98,99], and has been reported to be reduced in nasopharyngeal SCC [100,101] and oral SCC [102]. miR-29 has been shown to target *COL1A2*, *COL3A1*, *COL4A1*, and *SPARC* [100,103] in nasopharyngeal SCC. miR-204 has been reported to be decreased in hypopharyngeal SCC [104,105] and targets IL-8 [106]. Finally miR-199b has been reported to be decreased in HNSCC [102,105,107] and targets *LAMC2* [108]. These observations suggest a significant role for miRNAs in regulation of pro-metastatic and inflammatory pathways in HNSCC, and further investigation into miRNA regulation of aberrantly expressed mRNAs will undoubtedly help illuminate the mechanistic underpinnings of HNSCC pathology.

## Protein Arrays in HNSCC

Because separate patient tumors rely differently on individual cellular signaling pathways, protein signaling has become an important research focus. A better understanding of aberrations within these pathways will provide predictive and prognostic information and may also identify drug targets. In order to capture the signaling activity in these networks, the activated, phosphorylated state of a protein can be quantified through Reverse Phase Protein Arrays (RPPA). This technique generates snapshots of a patient’s cellular signaling network and, unlike gene expression analysis, will provide direct insight into post-translationally modified protein expression [109,110]. RPPAs are created by spotting denatured cellular lysate directly onto a nitrocellulose slide through a dilution curve. Plating multiple other samples and controls through RPPA allows a high throughput to be analyzed simultaneously with extremely sensitive analyte detection [109]. Validation is performed through Western blot and immunohistochemistry.

Studies investigating signaling pathways in HNSCC have enhanced our understanding of potential biomarkers and targeted therapies. Frederick *et al.* used RPPAs to examine 60 protein endpoints within previously untreated 23 HNSCC biopsy specimens and found 17 proteins decreased and 18 proteins elevated. The most significant protein elevations in tumor were checkpoint kinase p-Chk 1 (Ser 345), p-Chk (Ser33/35), eukarytotic translation initiation factor 4E-binding protein 1 p-4E-BP1 (Ser65), protein kinase C p-PKC zeta/iota (Thr410/T412), p-LKB1 (Ser334), inhibitor of kappaB alpha p-I*k*B-*α* (Ser32), eukaryotic translation initiation factor4E p-eIF4E (Ser209), p-Smad2 (Ser465/67), insulin receptor substrate 1 p-IRS-1 (Ser612), p-MEK1/2 (Ser217/221), and total PKC iota [111]. Specifically, PKC iota protein plays a major role in upregulating the NF-κB pathway and provides new information for potential treatment. Its increased activity and expression is also seen in 70% of primary and squamous subtype non-small cell lung cancers [111]. However, the tissue in this study was surgically resected HNSCC tumor specimens and matched adjacent nonmalignant tissue, which raises some concern about the effects of field cancerization in the adjacent “normal” tissue.

Similarly, Wheeler et al. applied RPPA to assess the prognostic value of EGFR Y992 and Y1068 within 67 HNSCC fresh frozen tumors from patients prospectively enrolled in surgery without an EGFR targeted agent [112]. The study used IHC to evaluate 154 patients in this cohort as well as 39 patients treated with chemoradiation involving EGFR targeted antibody cetuximab [112]. Elevated expression of total EGFR and phosphorylated EGFR PY1068 were independently significantly associated with reduced survival in the surgery cohort. STAT3 signaling downstream of EGFR PY1068 may be significant, and EGFR PY1068 had prognostic value in the HPV-negative cohort [112]. Tumor EGFR levels by IHC were associated with survival while EGFR levels by RPPA were not, which is speculated to be due to inherent differences in antibody and assay performance [112,113].

In our laboratory, Pernas et al. used RPPAs to investigate the effects of a drug targeting the epidermal growth factor receptor (EGFR), gefitinib, in HNSCC signaling pathways [30]. We tested p-EGFR (Tyr1068), p-AKT (Ser473), p-ERK(Thr202/204), p-RelA/p65(Ser536), p-STAT3(Tyr703), and each respective phospho-protein’s total protein level by RPPA in two HNSCC cell lines treated with EGF and gefitinib. We then validated the results obtained by RPPA with results previously obtained using Western blot and ELISA, observing similar trends in EGF activation or gefitinib inhibition of EGFR and downstream molecules. In addition, we performed RPPA on tumor lysates procured from 10 patients prior to gefitinib therapy, and 7 days after gefitinib treatment. Consistent with immunohistochemistry data, a broad decrease in RPPA staining of EGFR and downstream signaling molecules (10 of 13 biomarkers) was observed in a responder patient after gefitinib treatment, including the molecules involved in the AKT, ERK, STAT3, and NF-κB pathways. In addition, increased staining in cleaved caspase 3 was observed only in the specimen from the gefitinib responder, consistent with the increase in apoptosis detected by TUNEL assay in the same tumor specimen. In contrast, in a molecular non-responder patient, increased p-MEK, STAT3, p-STAT3, and p-NF-κB, were observed without increase in cleaved caspase 3. We concluded that the activation status of signaling components of downstream pathways of EGFR such as AKT, ERK, STAT3, and NF-κB contributed to the sensitivity and could serve as potential biomarkers of gefitinib in HNSCC patients [30].

## Bioinformatics and Systems Biology Analyses

The availability of massive datasets from large-scaled high throughput technologies creates the challenge of how to effectively analyze, integrate and interpret the data to reveal biological significance. Development of more sophisticated bioinformatics tools has become an effective approach to dissecting the complexity in biological functions behind the data generated from high throughput analyses. Gene expression is not a random event, but rather regulated by a coordinated operation of transcriptional and epigenetic factors in specific ways (activation or inhibition). Uncovering the combinatorial regulation among these factors is critical to understanding molecular mechanisms underlying cancer development and progression. Systems biology modeling of multi-dimensional resources and reconstruction of gene regulatory networks provides a solution to the complexity of information created with new technologies [114–116]. Previously, we employed a combined computational and experimental approach to determine two distinct gene signatures associated with *TP53* mutation status and NF-κB regulatory activity [18]. The analysis revealed that transcription factors TP53, NF-κB, and AP1 are important determinants of the heterogeneous pattern of gene expression in HNSCC, while STAT3 and EGR1 may broadly enhance gene expression levels in HNSCC cells. Furthermore, we have experimentally validated these transcription factors, NF-κB, TP53, AP1, STAT3 and EGR1, in modulation of gene expression in HNSCC cells [17,26,33,117,118]. Following this study, we applied a statistical method called COGRIM (based on Bayesian hierarchical model with Gibbs sampling) that was able to integrate heterogeneous data to capture transcription factor-gene associations [19]. We identified three sets of NF-κB regulons consisting of 748 target genes and the distinct signaling pathways of HNSCC cell subgroups associated with different *TP53* mutational status [19]. The predicted NF-κB target genes were experimentally validated by modulation with TNF-α or siRNA for RelA and NFκB1, and by demonstration of binding activity of the two NF-κB subunits to the promoter oligonucleotides [19].

To unravel NF-κB, AP1, p53, STAT3 and EGR1 regulatory interactions, we developed a integrative model that utilizes matrix decomposition under constraints of sparseness, which combines gene expression profiling and binding data of multiple regulators (transcription factors and miRNAs) for inferring gene regulatory networks [119]. Using this method, two transcriptional regulatory programs of seven key transcription factors (NF-κB, AP1, CEBPB, EGR1, TP53, SP1 and STAT3) were defined in two types of HNSCC cell lines (wild type and mutant *TP53* status). Ten target genes (*CDKN1A, CSF2, ELF3, FBXL11, IGFBP3, IL6, NDRG1, PTGS2, SERPINE1* and *TOB1*) were shared by the networks of both wild type and mutant *TP53* cell lines [119]. Furthermore, it is known that two miRNAs, oncogenic miR-21 and tumor suppressor miR-34 family are aberrantly expressed in HNSCC [84,85,87] and that they target p53 or NF-κB pathways [120–123]. We performed the newly developed bioinformatics model to infer gene networks co-regulated by NF-κB, p53, miR-21 and miR-34ac in HNSCC cell lines [119]. Interestingly, several genes in the network are related to metastatic processes, such as angiogenesis (*IL8*), cell adhesion (*SPP1* and *TNC*), and proteolysis (*MMP1* and *PLAU*). They are in accordance with the meta-analysis of microarray data of HNSCC tissues [45]. We further constructed similar transcription factor-miRNA networks by analyzing gene expression microarray data from metastatic and non-metastatic tissues in hypopharyngeal [124] and oral cancers [125]. As shown in Table 1, 21 genes from metastatic hypopharyngeal cancer and 41 genes from metastatic oral cavity cancer, including 9 shared, were identified as the targets of NF-κB, TP53 and the two miRNAs [119]. Among those genes, 11 from hypopharyngeal and 13 from oral cavity are overlapped with genes identified in the networks of HNSCC cell lines [119]. There are 4 genes, *MMP1, PLAU, SPP1* and *TNC,* identified in the microarray meta-analyses of both primary tumor and metastasis [45], supporting their biological significance, as well as the consistence of studies between cell lines and patient tissues. These computational and bioinformatics pipelines have provided important tools for showing the cross-regulation among NF-κB, TP53, and miRNAs, which may provide insights into the complex regulatory mechanisms underlying HNSCC development.

## Conclusion

The accumulation of genetic alterations and functional defects in multiple pathways leads to cancer development. Recent high throughput sequencing data further confirm that HNSCC is among the cancer types with the highest genomic variation [126]. Given this information, it is unlikely that durable responses can be achieved in most HNSCC patients using any single “magic bullet” therapy, as is the case for chronic myeloid leukemia patients receiving BCR-ABL inhibitors [127]. However, in order to overcome the complexity of the HNSCC genetic landscape, the use of high throughput technologies to generate unbiased global analysis of genetic and phenotypic defects, as well as bioinformatics to integrate information from large datasets derived from multiple sources, can help to expedite and streamline our ability to deepen the understanding about this disease process. The Cancer Genome Atlas project (http://cancergenome.nih.gov) focusing on HNSCC is such a collective effort, which has recently completed high throughput sequencing with different platforms and protein arrays of more than 300 HNSCC specimens. This project will provide more precise and unbiased genome-wide analysis of genetic and phenotypic variations of HNSCC. Currently, we are in an era capable of dissecting cancer genotypes and phenotypes in a more global and comprehensive way. Together with previously validated experimental results, these large sets of data provide a unique opportunity and challenge to translate genetic information into clinically useful interventions to benefit HNSCC patients.

## Figures and Tables

**Table 1:** NF-κB, p53, miR-21 and miR-34ac targeted gene programs of HNSCC tissues.

Cancer location	Genes in the network
Hypopharyngeal	*ALOX12B, CCL4, CD48,* ***IGFBP3****,* ***IRF4, LAMB3,*** *LDHA, PCBP1,* ***S100A2****,* ***SFN****,* ***SPP1****, TMEM109*
Oral cavity	*ARHGAP1,* ***ASS1****, BCL2, BHMT, BMP4, CEP57, COL1A2, CPEB3, CR2,* ***CSF1****, CXCR5, DNAJC16, FAS, GNLY, GPR64, GZMB, HNRNPK, IER3, IFNB1, MMP9, NFKB2, NOD2,* ***PERP****, PLA2G4A,* ***PLAU****, PSMA2, SEMA4C,* ***SERPINE1, SERPINF1****, STAT4,* ***TGFBI, TNC***
Both locations	*ALDOA,* ***IL1B, IL6, IL8, MMP1****, PTX3, SELE,* ***TP63****, TPM1,*

The target genes of transcription factors and miRNAs were predicted using the matrix decomposition-based method, based on microarray datasets of hypopharyngeal [124] and oral [125] metastatic vs. non-metastatic tumor tissues. Genes are presented as uniquely identified in hypopharyngeal cancer or oral cancer, or as appearing in both locations. Genes in bold are consistent with those genes differentially expressed and predicted to be under the regulation of NF-κB and TP53 in HNSCC cell lines with different *TP53* mutation status [18,19].
